# Sex differences in the psychometric properties of the Center for Epidemiological Studies–Depression (CES-D) Scale in older Filipinos

**DOI:** 10.1371/journal.pone.0286508

**Published:** 2023-06-14

**Authors:** Sanny D. Afable, Grace T. Cruz, Yasuhiko Saito

**Affiliations:** 1 Population Institute, University of the Philippines, Diliman, Quezon City, Philippines; 2 College of Economics, Nihon University, Tokyo, Japan; 3 Economic Research Institute for ASEAN and East Asia, Jakarta, Indonesia; University of Perugia: Universita degli Studi di Perugia, ITALY

## Abstract

The literature has yet to fully account for sex differences in the symptomatology and measurement of depressive symptoms, especially in developing settings like the Philippines. Thus, we established the factor structure and assessed the reliability of the 11-item version of the Center for Epidemiological Studies–Depression (CES-D) Scale for assessing depressive symptoms in older Filipino men and women. Using cross-sectional data from 5,209 community-dwelling Filipinos aged 60 and above from a nationally representative survey, Confirmatory Factor Analysis (CFA) and methods in Item Response Theory (IRT) were applied to provide complementary insights into the properties of the scale and its individual items. CFA supported the multidimensionality of the scale. The scale is also sex-invariant, but the relationship between the subfactors and the higher-order factor may differ between men and women. In addition, findings from IRT analysis confirmed the overall utility of the CES-D scale, but positively stated items were found to be internally inconsistent with the rest of the scale. The scale is desirably informative for assessing more severe symptoms, although sex differences were found in the precision of individual items. In general, the 11-item CES-D Scale is an adequate multidimensional tool for assessing moderate to severe depressive symptoms in the older population, especially in older men.

## Introduction

Despite being a global public health concern, mental health is an understudied aspect of the Philippines’ fast-aging population [1]. Depression is particularly a major area of concern given that it is the single largest factor contributing to global disability [2]. A strong body of evidence points to the comorbidity of depression with a range of other illnesses, such as neurological diseases and physical disorders, therefore affecting medical outcomes and increasing associated costs [3, 4].

To date, little is known about older Filipinos’ depressive symptomatology. While late-life depression shares core symptomatology with depression in the younger population such as chronic sadness and loss of interest, it is characterized by symptoms that are more distinct in the older population [5], including anhedonia or lack of pleasure, somatic symptoms such as musculoskeletal pain and peripheral body changes, and cognitive symptoms like problems in concentration and memory [5, 6]. Depressive symptoms may also vary across cultural contexts. Not feeling “happy” and not “enjoying life,” for instance, may not necessarily indicate an underlying depression, as demonstrated in Asian populations [7, 8].

These considerations merit closer examination when using standardized psychological instruments to measure depression. In the Philippines, two nationally representative surveys measured depression in older persons using shorter forms of the Center for Epidemiological Studies–Depression (CES-D) Scale, a widely used self-report tool for assessing depressive symptoms in the general population [9]. In studies using these surveys, simple sum-scoring was done to summarize depressive symptoms [10, 11], implicitly assuming scale unidimensionality and equal contributions of each item. Without proper validation of this scale, however, its application in older Filipinos remains in question.

Like any psychological instrument, the CES-D Scale must be particularly evaluated in terms of its structural validity, or “the degree to which the scores of [the instrument] are an adequate reflection of the dimensionality of the construct to be measured” [12]. Different factor scores, on the other hand, are established in the literature across varying populations [13–15]. Second, it must be evaluated whether the relative contribution of each item or factor in defining depression and the resulting scores are comparable across demographics. In particular, the overwhelming evidence of sex differentials in the prevalence of depressive orders and severity of depressive symptoms calls for an examination of possible sex differences in the presentation and measurement of depression [16–19].

Finally, the scale’s reliability must be assessed. In numerous applications, the CES-D Scale has been found to have high internal consistency, based in part on popular measures under Classical Test Theory (CTT), such as the widely cited Cronbach’s alpha (**α**). It has been demonstrated, however, that **α** does not relate to the internal structure of the test at all and may underestimate reliability [20]. In general, the weakness of CTT-based measures of reliability like **α** is that they are dependent both on the instrument and the sample characteristics [21]. Alternative ways of assessing reliability, such as factor analytic methods (e.g., McDonald’s omega, **ω**) and item response theory (IRT), are therefore suggested [21].

Against these contexts, this study assessed the psychometric properties of the CES-D Scale in older persons in the Philippines. It aimed 1) to establish the structural validity of the CES-D Scale, particularly its factor structure and measurement invariance across sex; 2) to assess the reliability of the CES-D Scale by examining its test performance and its item characteristics, and 3) examine sex differences in the presentation and measurement of depressive symptoms in late life.

## Materials and methods

### Data

This study is a secondary data analysis of the baseline survey of the 2018 Longitudinal Study of Ageing and Health in the Philippines (LSAHP), a longitudinal, nationally representative, and multi-actor study of community-dwelling older Filipinos aged 60 and above. The LSAHP study design was approved by the University of the Philippines Manila Research Ethics Board Review Panel 2 and was performed in accordance with relevant guidelines and regulations. Written informed consent was obtained from the respondent [22].

Employing a multistage sampling design, the baseline survey was conducted through face-to-face interviews from October 2018 to February 2019, and it oversampled those aged 70–79 by a factor of two and those aged 80 and above by a factor of three to ensure sample adequacy for follow-up surveys. Sampling weights should therefore be applied onto the analyses [22].

From a target sample of 6,335, a total of 5,985 individuals (94%) participated in the LSAHP, of whom 5,209 (87%) were eligible for an interview on account of physical and cognitive fitness.

### Measurements

Respondents were interviewed using the 11-item 3-response category CES-D Scale, which Kohout et al. derived from the original 20-item 4-response category scale [23] and was used in several studies [2, 10, 11]. Of the 11 items, two are positively stated, i.e., “You felt happy” and “You enjoyed life,” while the rest of the items express negative feelings. Respondents were asked to rate how often they felt these symptoms in the past seven days based on a three-response scale: 0 –Rarely/Not at all, 1 –Sometimes, and 2 –Often.

### Statistical analysis

This study employed two methods for assessing the psychometric validity of the CES-D Scale, namely, confirmatory factor analysis (CFA) and item response theory (IRT). Although both classes of methods are cut from the same cloth [24], they provide different but complementary and more comprehensive insights into scale properties.

First, CFA allows for an evaluation of structural validity, or how well the scale reflects the dimensionality of the underlying construct. CFA is a dimension reduction technique that is driven by prior knowledge of the underlying dimensions [25]. In this study, CFA was performed to compare the fit of five factor structures that are theoretically and empirically supported in the literature, namely, the unidimensional model [26, 27]; the correlated two-factor model consisting of the positive affect and the “negative” affect [15, 28, 29]; the correlated three-factor model that distinguishes the interpersonal affect [29, 30], and the correlated four-factor model that further breaks down the negative affect into the depressed affect and somatic retardation, just as originally conceptualized [9, 13, 25, 31]. Finally, the second-order factor model suggests that a higher-order factor explains the interrelationship between the four first-order factors [30].

In this study, CFA is conducted through robust weighted least squares (WLSMV) estimation. The WLSMV method is designed for ordinal data such as those derived from tools like the CES-D Scale which violates the multivariate normality assumption for ordinary factor analysis procedures [25]. This approach has been shown to be less biased than robust maximum likelihood estimation given adequate sample size [32]. Model fit was evaluated in terms of the following indices, namely: (a) the chi-square (χ2 > critical value); (b) the standardized root mean square residual (SRMR < 0.08); (c) the root mean square error of approximation (RMSEA < 0.06); (d) the comparative fit index (CFI > 0.95); and the Tucker-Lewis Index (TLI > 0.95) [25].

Once the factor structure had been established, measurement invariance across sex was evaluated by comparing the fit of increasingly constrained models through likelihood ratio testing. The configural invariance model only imposes the same factor structure for both men and women. The configural invariance model is then compared with the metric invariance model, which additionally assumes that the unstandardized factor loadings are the same for both groups, i.e., the latent constructs have the same meaning across sex. Finally, scalar invariance assumes that on top of having the same factor structure and equal factor loadings, the group means, e.g., factor scores resulting from the factor structure, can be directly compared [33].

To examine the scale’s reliability, we first calculated two well-known measures of internal consistency, namely, Cronbach’s **α** and McDonald’s Omega hierarchical (**ωH**). Both measures range from 0 to 1, with values close to 1 suggesting high internal consistency. Unlike **α** which only takes interitem covariance, the **ωH** is a factor-analytic measure and can account for the scale’s hierarchical factor structure [34].

Meanwhile, methods in item response theory (IRT) were also applied to evaluate the internal consistency and item characteristics of the CES-D Scale. Reframing the entire notion of reliability, IRT allows for an evaluation of the precision of the test in general and its individual items in particular across the continuum of latent ability (represented by θ), or in this case, the severity of depressive symptoms. The IRT enables an evaluation of the instrument at the item level without being affected by the sample’s characteristics. Despite its advantages, only a handful of studies have applied methods in IRT in assessing the CES-D Scale, whose item utility can vary [35].

To perform the IRT analysis, a graded response model (GRM) was fit for each sex. The GRM is suitable for ordered categorical responses and accommodates multidimensionality [35]. Positively stated items were first inverted because an assumption of GRM is that higher categories correspond with higher trait levels, or in this case, the severity of depressive symptoms, represented by θ. It has a mean of 0 and a standard deviation of 1, and its values may theoretically range between -∞ to +∞. The values of θ for depression typically range from -6 to 6, with values closer to -6 indicating less severe depression and values closer to 6 indicating more severe depressive symptoms [36]. Following Bean & Bowen [24], the fit of the GRMs were examined using the following fit indices: C2-RMSEA ≤ 0.06 and SRMR ≤ 0.05.

Resulting from the GRM, we generated and examined the scale and item characteristics based on the option characteristic curves (OCCs), item information curves (IICs), and the test information curve (TIC). For a given item, the OCC plots the probability of selecting a particular response category across the range of values of θ. It is desired that the OCCs reflect the diversity of responses across θ for a given item. Meanwhile, the IICs summarize the individual contribution of each item to the scale. The IIC of a given item should be able to cover a range of θ as an indication of item reliability. The sum of all IICs is the total information, which measures the overall scale reliability.

CFA was performed using the *lavaan* (Latent Variable Analysis) package in R [37], while IRT analysis was performed using the ‘MIRT’ package in R [38]. P-value < .05 was considered statistically significant throughout the analysis.

## Results

### Structural validity

Results of CFA do not support a unidimensional factor solution for the 11-item scale, given that the fit indices of the one-factor model failed to reach the suggested cutoffs for the fit indices (Table 1). The two-factor model had an acceptable fit, while the three-factor model met all the cutoffs for the fit indices. Of all the factor models, the four-factor solution was the best fitting model, yielding the lowest SRMR and the highest CFI and TLI.

**Table 1:** Goodness-of-fit indices of hypothetical factor structures of the Center for Epidemiological Studies–Depression (CES-D) Scale.

Model	χ2 (df)	RMSEA (90% CI)	SRMR	CFI	TLI
Unidimensional	577.0* (66)	.039 (.036,.041)	0.112	0.827	0.856
Correlated two-factor	228.6* (66)	.022 (.019,.025)	0.082	0.945	0.954
Correlated three-factor	145.8* (65)	.015 (.012,.019)	0.065	0.973	0.977
Correlated four-factor	132.7* (62)	.015 (.011,.018)	0.063	0.976	0.979
Second-order factor	136.6* (64)	.015 (.011,.018)	0.065	0.975	0.979

*Note*: robust fit indices: RMSEA–Root Mean Square Error of Approximation; SRMR–Standardized Root Mean Square Residual; CFI–Comparative Fit Index; TLI–Tucker Lewis Index

* *p* ≤ .001.

Except for positive affect items which correlated weakly with other factors, the subfactors of the four-factor model were highly correlated, with correlations ranging from 0.65 to 0.86. This indicates that a higher-order factor explains these first-order factors. Indeed, the higher-order factor solution is a valid factor model, as indicated by its fit indices that are highly comparable with that of the four-factor model. The advantage of the second-order factor model is that instead of treating the CES-D Scale as consisting of multiple subfactors, a generalized depression factor can represent the severity of depressive symptoms and subsequently be used for deriving a single set of latent scores [31]. For this reason, the higher-order factor model was deemed the best factor structure of the CES-D Scale and subjected to tests for invariance.

**Fig 1:**
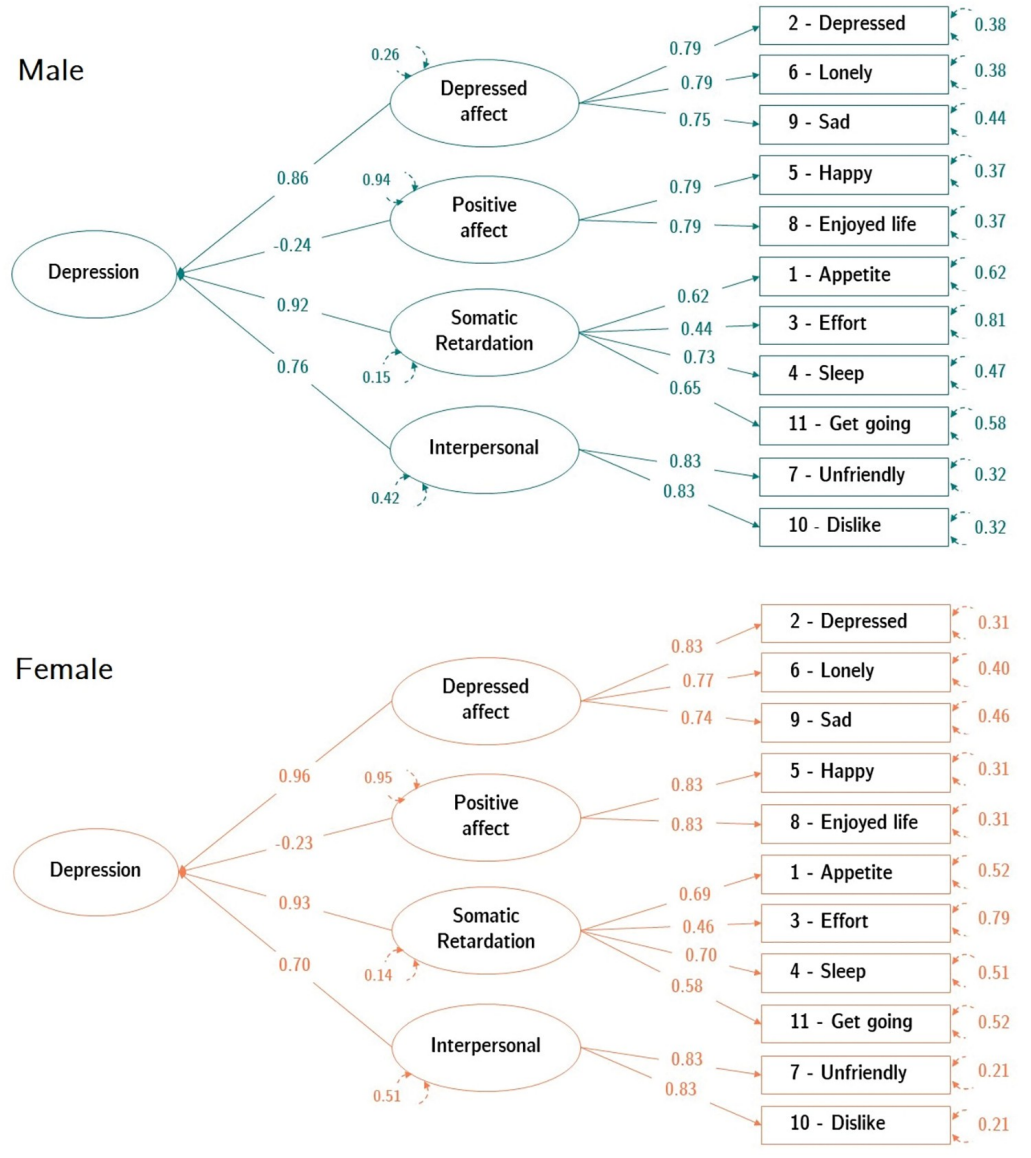
Path diagram of the higher-order factor model and its completely standardized estimates, by sex. *Note*: The numerical values of the lines or paths refer to the factor loadings, or the degree of correlation between the indicator and the factor, with absolute values close to 0 indicating low correlation and values close to 1 indicating high correlation. Higher absolute factor loadings correspond with lower measurement errors, given by the values in circles.

When freely estimated, the standardized factor loadings of the higher-order factor structure are shown in Fig 1. The same pattern holds across sex: all CES-D Scale items had high loadings onto their corresponding subfactors, except for item 3, which has a factor loading less than 0.50. In terms of the latent factors, the positive affect had a low correlation with the higher-order factor. On the other hand, the depressive and somatic symptoms had the highest correlation with the higher-order factor, but they present different patterns by sex in terms of the standardized loadings. The higher-order factor correlated the highest with somatic retardation in men and with the depressed affect in women. Despite these differences, the tests of invariance—which are based on the unstandardized factor loadings—indicate that the higher-order factor structure is metric- and scalar-invariant across sex (Table 2).

**Table 2:** Tests for measurement invariance across sex of the higher-order factor structure of the Center for Epidemiological Studies–Depression (CES-D) Scale.

Model	χ2 (df)	RMSEA (90% CI)	SRMR	CFI	TLI	Decision
Configural invariance	177.57 (130)	.012 (.007,.016)	0.071	0.985	0.987	
Metric invariance	175.71 (92)	.019 (.014,.023)	0.072	0.974	0.968	Accept
Scalar invariance	186.31 (98)	.019 (.014,.023)	0.070	0.972	0.969	Accept

*Note*: robust fit indices: RMSEA–Root Mean Square Error of Approximation; SRMR–Standardized Root Mean Square Residual; CFI–Comparative Fit Index; TLI–Tucker Lewis Index

### Reliability of the CES-D Scale

The value of Cronbach’s **α** is 0.74, just above the conventional 0.70 cutoff for it to be considered acceptable [39]. This is also lower than the computed α in other studies [15, 40]. But as previously mentioned, **α** can misrepresent instrument reliability in general. We thus computed omega values for the four-factor and higher-order factor models, respectively. The omega values of the four first-order factors and the higher-order factor were satisfactory (**ωH**_Depressed_ **=** .74; **ωH**_positive_ **=** .69; **ωH**_interpersonal_ **=** .64; **ωH**_somatic_ **=** .60; **ωH**_higher_ **=** .89), and somewhat consistent with the results of one study in China [34].

When using all the 11 items, the GRMs for male and female subsamples had a less than desirable fit (male: RMSEA = 0.12, SRMSR = 0.10; female: RMSEA = 0.11, SRMSR = 0.10), but the model substantially improved when the two positive affect items were excluded (male: RMSEA = 0.06, SRMSR = 0.07; female: RMSEA = 0.04, SRMSR = 0.08). Since the predictive validity of the model is not the focus of this study, however, the GRM for the full 11-item scale is presented here to allow for the examination of the individual items, including the poorly fitting positive affect items.

**Fig 2:**
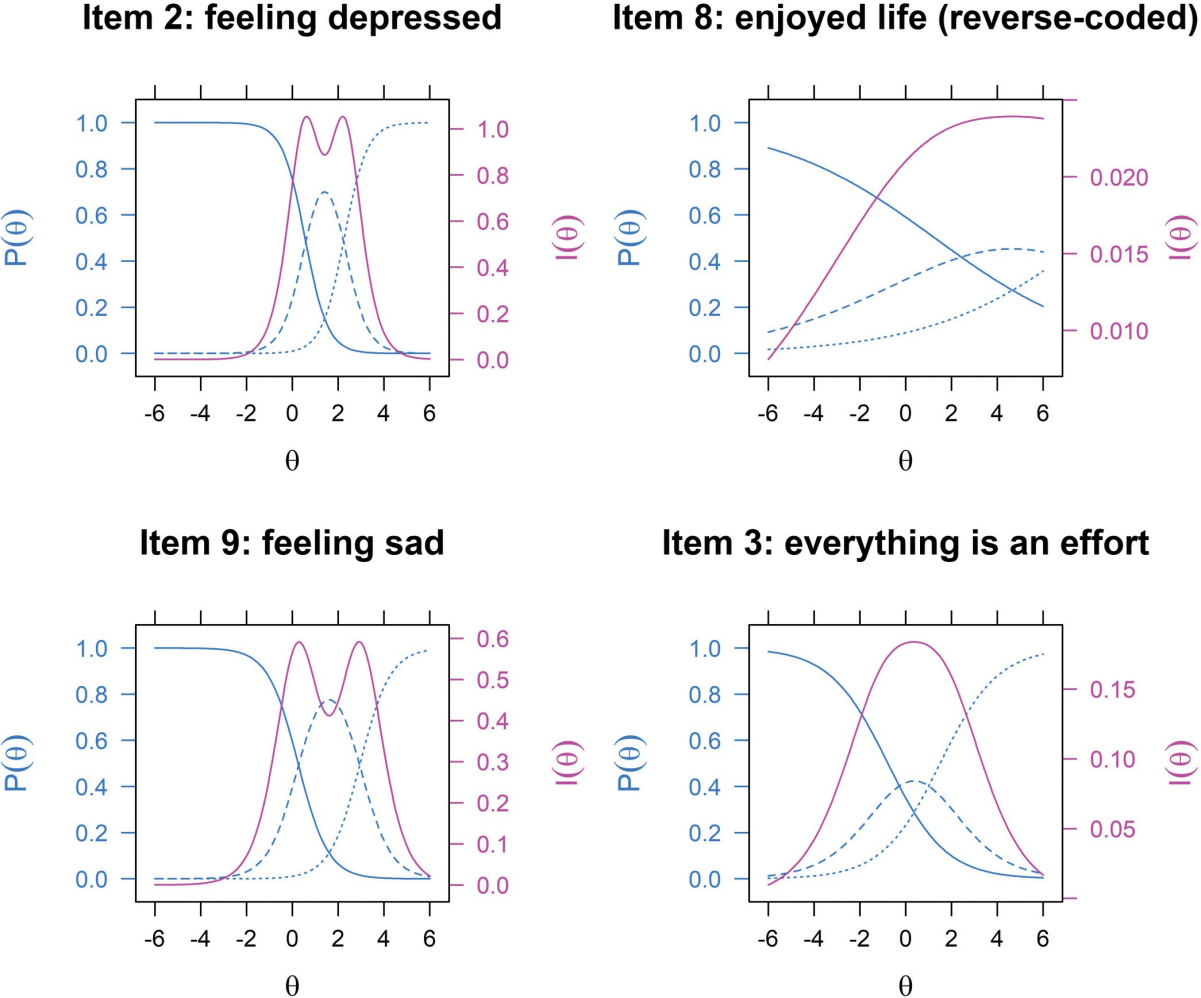
Option characteristic curves and item information curves for selected items from the 11-item Center for Epidemiological Studies–Depression (CES-D) Scale, male. *Note*: The blue lines are the option characteristic curves, P(θ), while the magenta lines are the item information curves, I(θ).

For the purposes of illustration, Fig 2 shows the OCCs and IICs for selected items using the male subsample. These items are compared due to their starkly contrasting characteristics. In item 2 (*depressed*), those who had exhibited severe depressive symptoms (at about θ ≥ 3) were more likely to endorse response category 2 (i.e., always felt depressed), thereby confirming the utility of this item in screening depression. Its high and narrow IIC also suggests that it can discriminate well between those with less and more severe depressive symptoms and thus provide precision to the scale. On the other hand, the responses to reverse-coded item 8 were dominated by category 0 (i.e., often enjoyed life) even at higher values of θ, wherein the probability of selecting any of the two higher categories was less than 0.4. Its IIC indicates that it provides an extremely low amount of information.

Items 3 (*effort*) and 9 (*sad*) also present contrasting properties. The OCC of the former indicates that the responses to it were polarized, that is, most older persons reported either never/rarely feeling that everything they did was an effort or always feeling this way, and the middle category (“sometimes”) was underutilized. In turn, item 3 was less informative than item 9, wherein the three response categories were fairly utilized and could thus discriminate between varying intensities of depressive symptoms.

**Fig 3:**
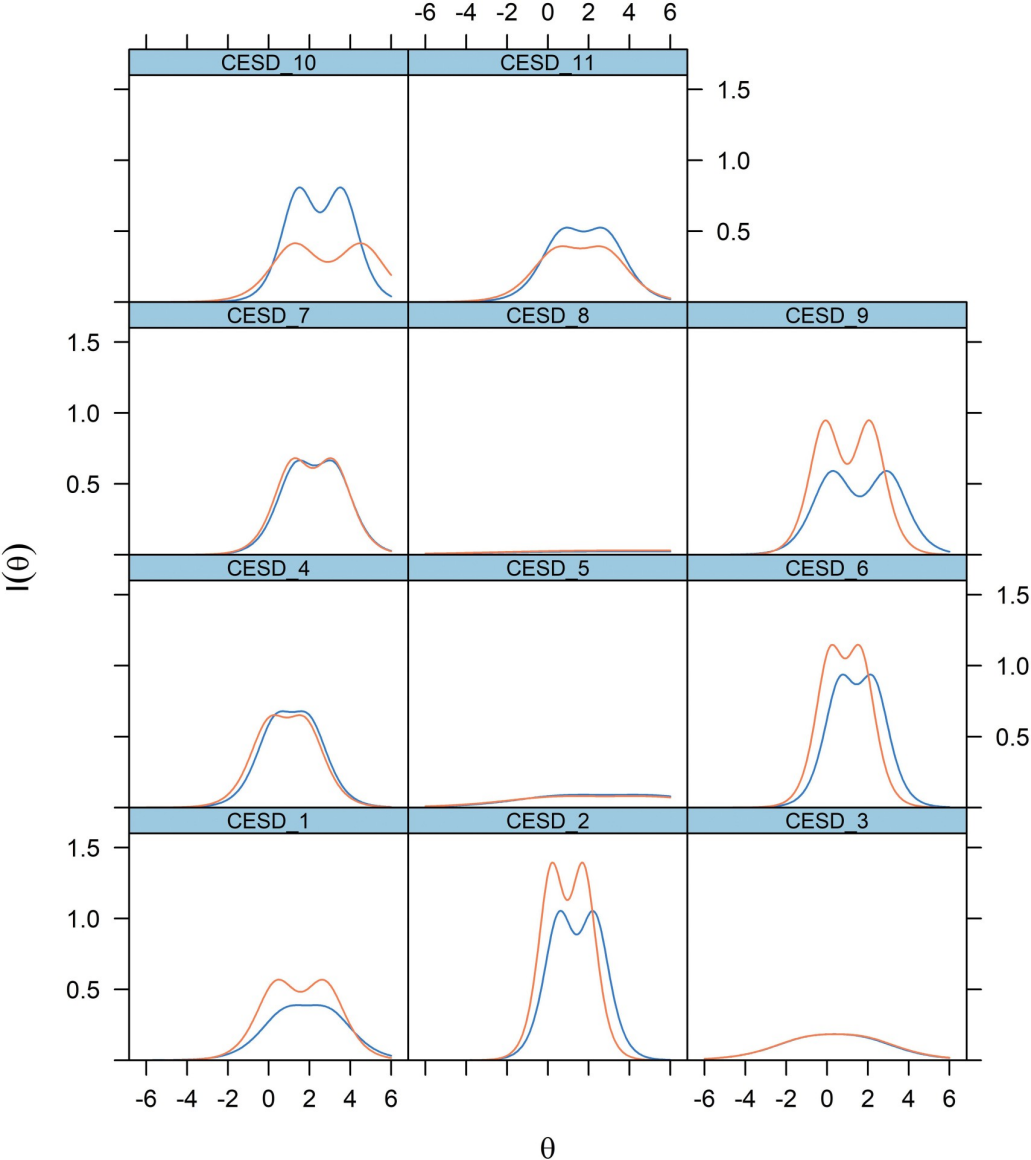
Item information curves of the 11-item Center for Epidemiological Studies–Depression (CES-D) Scale, by sex. *Note*: 1 –poor appetite; 2 –feeling depressed; 3 –feeling that everything was an effort; 4 –restless sleep; 5 –feeling happy; 6 –feeling lonely; 7 –feeling that people are unfriendly; 8 –enjoyed life; 9 –feeling sad; 10 –feeling that people dislike you; 11 –could not get going.

**Fig 4:**
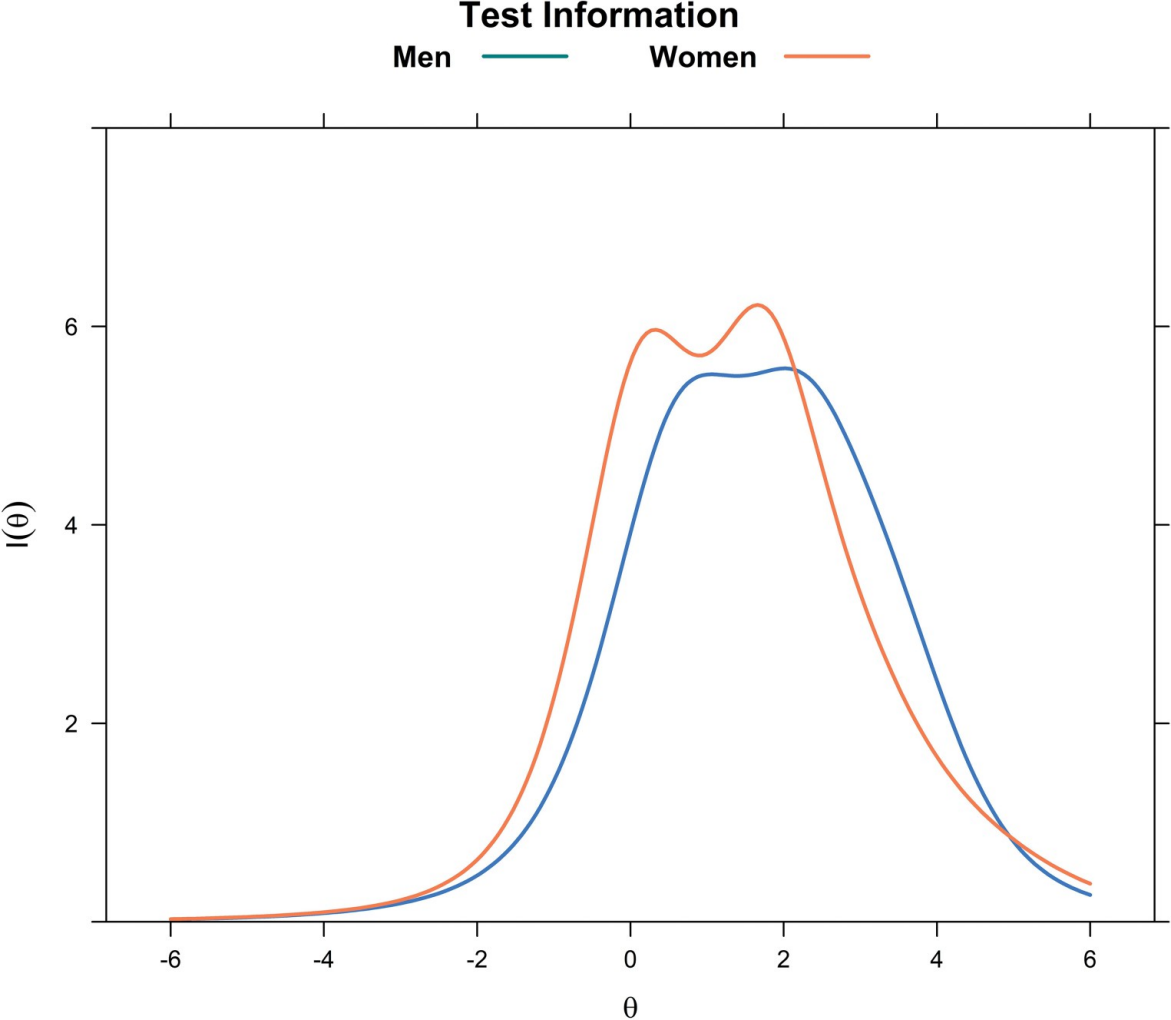
Test information curves of the 11-item Center for Epidemiological Studies–Depression (CES-D) Scale, by sex.

The IICs of all items for men and women subsamples are given in Fig 3. For both men and women, items 2 (*depressed*), 4 (*restless sleep*), 6 (*lonely*), 7 (*unfriendly*), and 10 (*dislike*) were the most discriminating and informative. In contrast, item no. 3 (*effort*) and the two positive affect items provided inadequate information. Item 9 (*sad*) was more informative in women, while item 10 (*dislike*) is more informative in men. In general, as Fig 4 shows, the CES-D Scale is more informative in men, providing maximum information for values of θ from 0 to 3 in men, and from -1 to 2 in women. Away from these values, the scale becomes less reliable.

## Discussion

This study provides many insights into the utility of the CES-D Scale in measuring depressive symptoms in older Filipinos using a nationally representative sample. First, we find that the CES-D Scale should be conceived as a multidimensional instrument for measuring the severity of depressive symptoms in older Filipinos. It measures four subdimensions of depression, namely, depressed affect, somatic retardation, interpersonal affect, and positive affect, as identified by Radloff [9]. There is no reason to believe that Filipinos do not make a distinction between their feelings and somatic complaints, as Fernandez et al. argued [29], given that the four-factor model yielded a better fit than the two-factor model. The four intercorrelated factors are further explained by a single higher-order construct of depression [30]. In any case, the poor fit of the unidimensional model and the disparate contributions of the scale items to operationalizing depressive symptoms evince that treating the scale items equally, as in sum-scoring, can lead to high measurement error. Although the practicality and accuracy of using factor scores vis-a-vis simple sum scores remain an issue in psychometric research [41–43], our results suggest that due consideration must at least be given to the scale’s multidimensionality when utilizing and interpreting sum scores.

Additionally, the high factor loadings of the depressed affect and somatic retardation on the higher-order construct indicate that they are the two most important sub-factors in explaining depressive symptomatology. This corroborates the whole body of work that finds depression as both an affective and physical condition in late life [44, 45]. Stratification by sex, however, revealed differences in how depression manifests in men and women. Somatic retardation had the highest correlation with the higher-order factor in men. In contrast, the depressed affect has the highest correlation with the higher-order factor in women. This finding is backed by the observation of Johnson et al. that the depressed affect accounts for the elevated depression scores of Canadian women [46]. Additionally, Thayer found that women are generally more cognizant of their emotions, but women with high depressive symptoms demonstrate greater emotional awareness and are more likely to ruminate than men who have similar levels of depression [47]. This difference, however, does not affect the invariance of the scale. It was demonstrated that for both men and women, the factor structure of the scale is the same, the set of unstandardized factor loadings are equal, and the sets of item intercepts are equivalent—indicating that the set of scores resulting from the higher-order factor structure has no apparent sex bias, consistent with other CFA studies [15, 18, 19].

Although the internal structure of the scale does not appear to vary by sex, the CES-D Scale and its individual items may provide varying levels of precision between men and women. Depressed affect items, namely items 2 (*depressed*), 6 (*lonely*), and 9 (*sad*) are more informative in women. Meanwhile, somatic items 11 (*get going*) and interpersonal item 10 (*dislike*) provide more information in men than in women for a range of symptoms severity, corroborating the earlier finding about the sex differential in the correlation of depressive affect and somatic retardation with the higher-order construct of depression. As James et al. argued, some items of the CES-D Scale may fail to adequately capture the phenomenology of depression in men, who may be more reserved in expressing their emotions [35].

Moreover, item-response analysis confirmed the utility of the depressed affect, somatic retardation, and interpersonal affect items in explaining depressive symptomatology. Except for item 3 (*effort*), all these items were informative and could discriminate well between those with low and high depression scores, especially items 2 (*depressed*) and 6 (*lonely*). It is worth noting as well that for both men and women, item 6 (*lonely*) is more informative than item 9 (*sad*) for a range of more severe depressive symptoms. This suggests that loneliness—an emotional state resulting from perceived social isolation, as opposed to the more general feeling of sadness—is a key feature of late-life depression, as previous studies have demonstrated [48, 49]. In fact, it is quite common for older people with depression to deny feeling sad [50], thus the relevance of other dimensions such as loneliness and somatic symptoms in characterizing late-life depression.

The positive affect had a weak correlation with the higher-order factor, which indicates that rarely feeling happy or not at all does not translate to feeling sad or depressed often. Its corresponding items also had inadequate performance, which is line with the results of one study that used non-parametric IRT [51]. It could be that the responses to the positive affect items are “contaminated” by other experiences, such as stress and anxiety [51]. Several studies also link such result to the tendency of Asians to suppress or underreport their positive emotions [7, 15, 52, 53]. The opposite pattern holds for older Filipinos, however, whereby an overwhelming majority stated that they “often” felt happy and enjoyed life—consistent with a few studies showing Filipinos’ exceptional tendency to rate themselves as happy [54, 55]. This unique finding suggests that positive feelings can be present even among Filipinos with more severe depressive symptoms. In the context of the CES-D Scale, the positive affect could only be obfuscating the measurement of depressive symptoms. Subsequent applications and development of the CES-Scale may rethink the inclusion of these items, as James et al. similarly recommended [35].

In any case, even in the presence of positive affect items, the CES-D Scale is demonstrated to have high internal consistency. The scale and individual items particularly provide a high amount of information among those with more severe depressive symptoms, especially among men, which is ideal for initial diagnostic screening for major depression. No such diagnostic tool that has locally validated cutoff scores is available in the Philippines, but future research on this area may consider the CES-D Scale.

While this study’s findings are generalizable only to the older Filipino population, they can inform the application of the CES-D Scale or the development of depression scales in other age groups in the Philippines. For one, depression’s multidimensionality—particularly the distinction between somatic and affective symptoms—may be a feature that cuts across other age groups, and is in line with current diagnostic criteria for major depressive disorder [56]. In addition, the study findings may be relevant to the older populations of other lower middle-income countries in Asia, whose socioeconomic contexts could make them more vulnerable to depression [35].

This study is not without limitations. The equivalence of items’ residuals of the CES-D Scale was not established, although residual invariance is hard to achieve in most cases and the residuals do not anyway affect the interpretation of scores [33]. The CES-D Scale was also translated into three local languages in the LSAHP, but this study did not account for possible differential effects of the translations on the responses to the scale.

## Conclusions

The CES-D Scale is a multidimensional and generally sex-invariant tool for assessing depressive symptoms in the older Filipino population. It is particularly useful for screening individuals with moderate to severe symptoms, especially in older men. Interpretation of derivative scores must account for sex differences in the levels of scale and item precision, which is reflective of the sex-specific presentations of late-life depressive symptoms.
